# Total Hip Arthroplasty for Bilateral Femoral Neck Stress Fracture: A Case Report and Review of the Literature

**DOI:** 10.1155/2019/2720736

**Published:** 2019-12-14

**Authors:** Kevin Moerenhout, Georgios Gkagkalis, G.-Yves Laflamme, Dominique M. Rouleau, Stéphane Leduc, Benoit Benoit

**Affiliations:** ^1^Orthopedic Surgery, Department of Surgery, Hôpital Sacré-Cœur de Montréal, 5400 Boul. Gouin O., Montréal, Québec H4J 1C5, Canada; ^2^Department of Orthopaedics and Traumatology, Lausanne University Hospital, Rue du Bugnon 46, CH-1011 Lausanne, Switzerland

## Abstract

Femoral neck stress fractures (FNSFs) can be treated conservatively or surgically, depending on initial displacement and patient condition. Surgical treatment options include internal fixation, with or without valgus osteotomy or hip arthroplasty, either hemi or total. The latter is mainly considered when initial treatment fails. A review of the literature shows that total hip arthroplasty (THA) is only considered as primary treatment in displaced fractures (type 3) in low-demand patients. We present a case of successive bilateral FNSF in a young active patient, where a THA was performed on one side, after failed internal fixation, and where it was chosen as primary treatment on the other side after failed conservative treatment.

## 1. Introduction

Stress fractures are infrequent lesions due to overloading and/or repetitive overuse and can occur at several sites, with the more common being the metatarsals, femoral/tibial shaft, and femoral neck. They can be subcategorized into insufficiency fractures when an abnormal bone fractures under normal stresses and into fatigue fractures when a normal bone fractures under abnormal stresses [1]. The physiopathologic substrate is the combination of an imbalance between bone remodeling capacity and repetitive stress. Endocrine, metabolic, and pharmacologic causes have been described.

Femoral neck stress fractures (FNSFs) represent 5% of all stress fractures in the general population and are encountered more often, up to 15%, in athletes [1]. More specifically, it is seen in long-distance runners and can be associated with the “female athlete triad” [2, 3]. In nondisplaced incomplete femoral stress fractures, treatment is nonoperative with non-weight-bearing and activity cessation. In complete fractures, or when conservative treatment fails, internal fixation by percutaneous screw fixation or dynamic hip screw (DHS) is applied. Other treatment possibilities are intertrochanteric osteotomy or hip arthroplasty, either hemi or total (THA). The latter is mainly reserved as a salvage option in cases where previous surgical treatment has failed. We present the case of a young patient who presented with femoral neck stress fractures on both sides within a two-year span. THA has been chosen as a salvage procedure after nonunion following internal fixation on one side and as the primary surgical treatment on the other side.

## 2. Case Report

**Figure 1:**
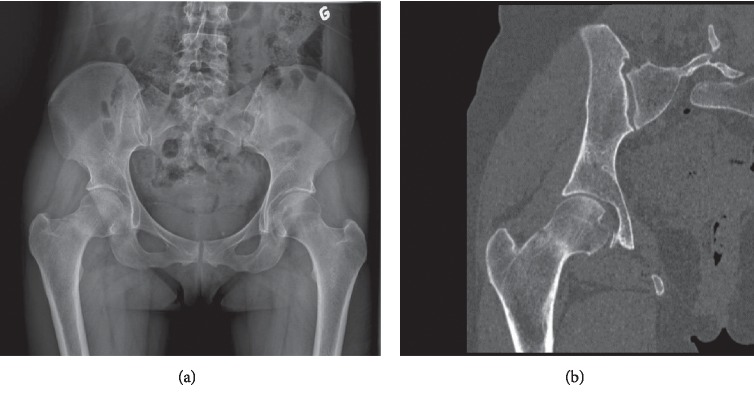
(a) AP pelvic X-ray showing a stress fracture of the right femoral neck. (b) Frontal view on CT scan of the right hip confirming the femoral neck fracture.

**Figure 2:**
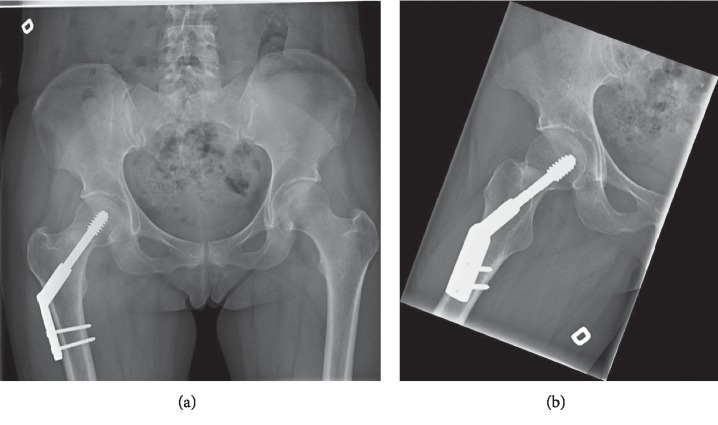
(a) AP pelvic X-ray and (b) lateral view of the right hip after dynamic hip screw (DHS), 6 weeks after surgery. The implant is in good position.

**Figure 3:**
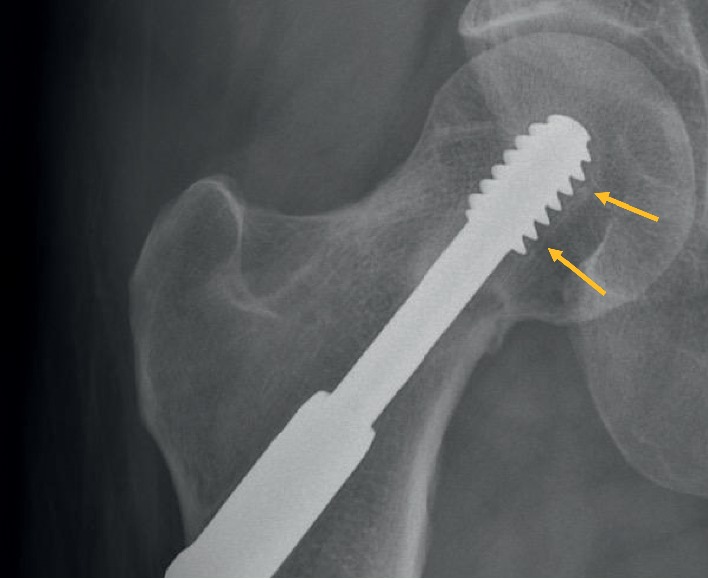
Right hip X-ray showing mobility around the DHS (arrows) and a fracture line on the compression side.

**Figure 4:**
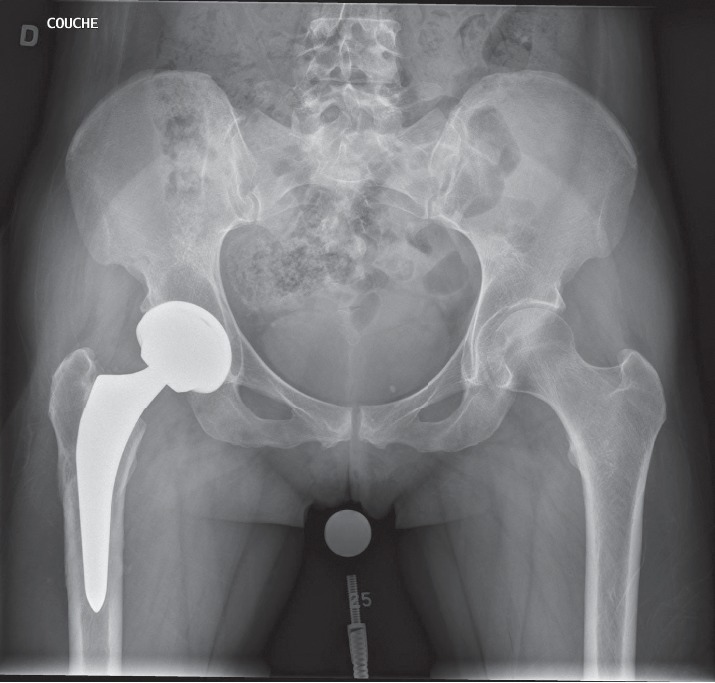
AP pelvic X-ray 6 weeks after right noncemented THA.

A 46-year-old female patient, who used to run three times a week (20 km/week), presented to our outpatient clinic in 2016 with right groin pain, which had begun a few days before without trauma. She was under treatment for multiple sclerosis since 2002, taking teriflunomide daily. Initial X-rays showed a dense line in the femoral neck, with lateral cortex disruption on the tension side. A stress fracture was suspected and confirmed on CT scan (Figures 1(a) and 1(b)). The patient underwent internal fixation by DHS with a good radiographic result (Figure 2). However, groin pain was still present after 1 year, disabling all sports activity. X-rays were taken, showing radiolucency around the cervicocephalic screw, probably due to interfragmentary mobility, and signs of nonunion, mainly on the compression side (Figure 3). A THA using a cementless stem and a press-fit cup (AMIS/Versafit, Medacta®, Switzerland) with ceramic on ceramic bearing was performed (Figure 4), without complications. Pathological exam of the retrieved femoral head showed avascular necrosis.

**Figure 5:**
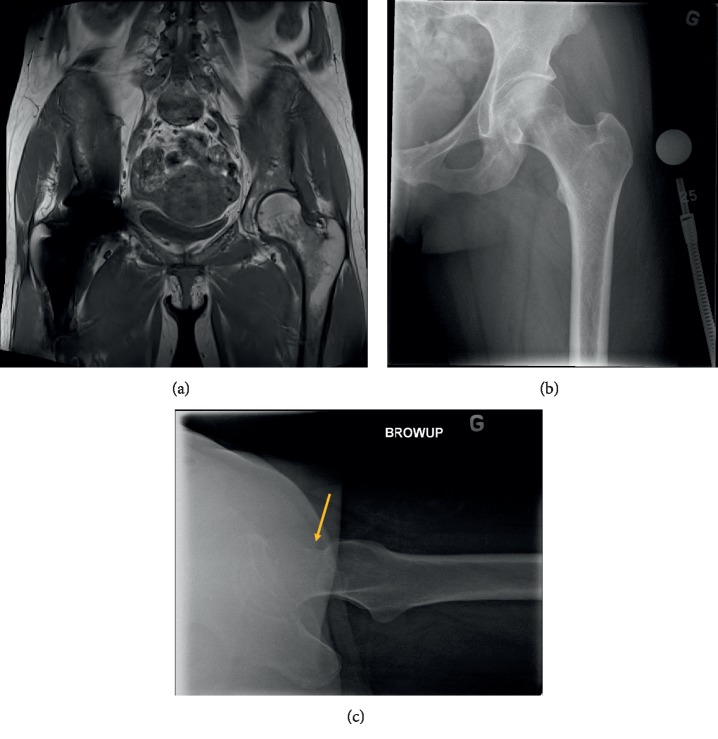
(a) Pelvic MRI showing bone marrow edema of the left hip on the compression side. (b) AP and (c) profile follow-up X-ray of the left hip showing a minimally displaced complete femoral neck fracture (arrow).

Two years later, aged 48 years, the patient returned because of pain in her left groin, with no traumatic event present in the previous days. Initial X-rays and CT scan were normal. Due to the history of the patient, non-weight-bearing was applied and an MRI was taken. This MRI showed bone marrow edema of the left hip on the compression side. Follow-up X-rays showed a minimally displaced femoral neck fracture of the left hip (Figure 5).

**Figure 6:**
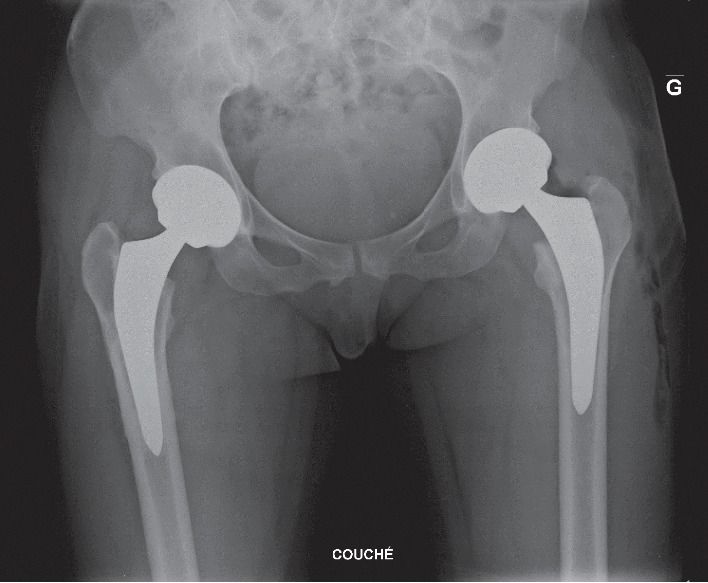
Postoperative pelvic X-ray after left total hip arthroplasty and three years after right total hip arthroplasty. Implants are in good position.

Blood samples and metabolic analysis (vitamin D, Ca, P, TSH, PTH, and VIH) were normal. Body mass index was normal, and “female athlete triad” syndrome was excluded as there were no menstrual abnormalities. Osteoporosis was excluded by DEXA scan. Four months of nonoperative treatment with non-weight-bearing protocol found the patient still in pain. X-rays confirmed a nondisplaced complete fracture of the femoral neck. After discussion with the patient, we opted for primary THA (Figure 6). The same combination of implants as the contralateral side was used. The patient was discharged from hospital one day after surgery with no adverse events, with a Harris Hip Score (HHS) of 94 and a Hip disability and Osteoarthritis Outcome Score (HOOS) of 85.6 for her left hip at 5 months postoperatively. HHS and HOOS were, respectively, 99.9 and 94.6 for her right hip at two years after surgery. The patient regained walking activity without support four weeks after surgery but has yet to regain all previous sports activity.

## 3. Review of the Literature

A medical literature search was performed in MEDLINE (PubMed) database. The keywords used were bilateral, femoral, neck, stress, fatigue, and fractures. Only papers published in English were considered. Radiology papers as well as papers with fractures due to trauma or located elsewhere than the femoral neck were rejected. Three case series and 36 case reports matched the search criteria and were retained for review.

Bilateral FNSFs are only described in case reports or minor case series, with various management and failure rates (Table 1). Miller has reported the first documented case of bilateral FNSF in the literature in 1950 [4]. Cases of bilateral femoral neck fractures, even simultaneous, have been reported in healthy military recruits [5–9] as well as in healthy nonathletes [10, 11]. Insufficiency or fatigue bilateral fractures of the femoral neck have been described in various conditions such as pregnancy [12]; bone metabolic diseases, caused by osteomalacia associated with coxa vara [13] or by celiac disease [14]; vitamin D deficiency [15]; and rare genetic syndromes like Marfan [16] and autosomal dominant osteopetrosis [17]. Other more common risk factors, such as steroid treatment [18, 19] and anorexia nervosa [20], have also been involved in cases of bilateral insufficiency femoral neck fractures. Bilateral FNSF has also been described as a very rare complication following simultaneous bilateral total knee arthroplasty [21].

Among the 46 patients with bilateral FNSF found in the literature, 23 were male and 23 were female, with a mean age of 35 years. Fifty-six percent were on compression, 18% were on the tensile side, and 26% were displaced. Twenty-three percent were treated conservatively; 56% were treated by internal fixation; 12% had an osteotomy; and eight (9%) out of 92 patients had a prosthetic implant, among them only two had a total hip replacement. Failure rate for internal fixation was 11.5%. Internal fixation methods used in those cases were screws in four patients and gamma nails in one patient, bilaterally. Two (10%) of the 21 patients with conservative treatment were eventually operated, with screws in one case and hemiarthroplasty in the other. One patient went on being treated conservatively using bisphosphonates, although the femoral neck fracture on one side was still visible in the X-rays at 24 months of follow-up postinternal fixation [22]. Failure rate for conservative treatment was three (14%) out of 21 patients.

No complications were recorded with the osteotomy treatment in those series nor with the prosthetic implants. Follow-up was inconsistent, ranging from 2 weeks to 15 years. No quality of life questionnaire or other outcome measuring score was reported in any of the case reports.

## 4. Discussion

**Figure 7:**
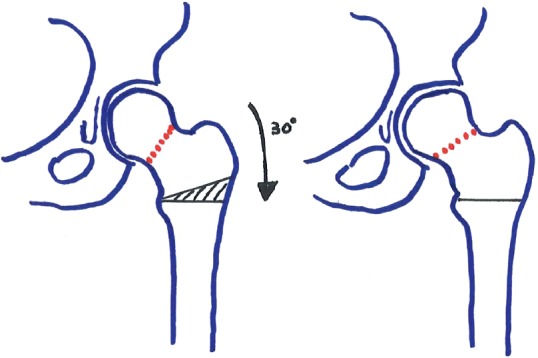
Valgus intertrochanteric osteotomy: by subtracting a 30° wedge on the lateral femur (black hatched), the vertical stress fracture (red hatched) on the left becomes 30° more horizontal on the right.

Different classifications exist regarding FNSF. Fullerton and Snowdy proposed a classification of FNSF in three types, based on biomechanics and degree of displacement: type 1 is a compression-sided fracture; type 2 is a tension-sided fracture; and type 3 is a displaced fracture [23]. If fracture lines are less than 50% of the neck, treatment of femoral neck stress fractures is nonoperative, consisting of non-weight-bearing and activity cessation. If compression or tension-sided fractures have fracture lines greater than 50% of the neck, percutaneous screw fixation must be strongly considered [23]. Type 2 stress fractures, as found in the right hip of our patient, often heal poorly and therefore require treatment that is more aggressive in order to avoid malunion, nonunion, or osteonecrosis, leading to hip osteoarthritis [24]. After nonunion following DHS on the right hip, we opted for THA. Other treatment options, such as valgus intertrochanteric osteotomy with DHS or blade plate fixation in order to compress and stabilize the stress fracture by converting shear forces to compressive ones, have been described with variable failure rates [25, 26]. Valgus intertrochanteric osteotomy permits a more horizontal reorientation of the stress fracture line by subtracting a wedge on the lateral femur (Figure 7). THA has given good results and more predictable failure rates.

We opted for primary THA on the patient's left hip once conservative treatment failed. This treatment option can be disputed. As the second femoral neck stress fracture was on the compression side, it would have had more healing potential than the contralateral tension-sided stress fracture. These two fractures are therefore not comparable in outcome. Although valgus intertrochanteric osteotomy was a valid therapeutic option age wise, we believed it presented a potential failure risk, resulting from avascular necrosis of the femoral head, given that this was the pathological finding on the contralateral side. In such a case, we would have been confronted with a more complicated primary THA requiring the extraction of material and a hip with altered biomechanics from the valgus orientation of the proximal femur. This was explained to the patient, but she refused to go through the same process with the risk of a potential failed internal fixation and preferred the option of THA. Another reason for her decision is that, as an independent worker, she needed to get rapidly back to work. Taking a longer leave of absence from work was no longer an option for her.

This case highlights the potential of primary THA in femoral neck stress fractures. We found only one case of bilateral femoral neck stress fractures treated by THA in the literature, but this case reported on a debilitated cerebral palsy patient [27]. To the best of our knowledge, this case is the first to report on a young active patient treated by THA for bilateral femoral neck stress fractures. Although this should not be the gold standard, it should be considered as a treatment option, especially in young high-demand patients who want to quickly regain unrestricted and painless mobility of their hips. Failure rate following internal fixation of femoral neck fractures in the nonelderly population has been reported as high as 59% [28]. Complications associated with treatment by internal fixation, such as avascular necrosis (12–86%), nonunion (10–59%), as well as early-onset hip osteoarthrosis, are avoided with the THA option [29]. Although all the risk factors associated with traumatic femoral neck fractures are well described in the literature, data about the incidence of these complications in femoral neck stress fractures are lacking. The incidence is dependent on the situation (tensile versus compression side), fracture line (complete versus partial), and initial displacement of the fracture. Potential complications associated with THA, such as infection or dislocation, must be considered and clearly explained to the patient. The incidence of infection and dislocation following THA for femoral neck fractures are probably the same as those in the general population, but we do not have the data to back this assumption. However, salvage THA after failed internal fixation of the neck fracture is associated with higher complication rates [30–32].

In cases of uni- or bilateral neck stress fractures, a metabolic and systemic assessment is mandatory in order to rule out a metabolic cause favoring the stress fracture. Menstrual abnormalities and eating disorders must always be investigated when femoral neck stress fractures are present. In the present case, blood and metabolic workup were normal. The patient had no risk factor other than running three times a week, for a total of 20 km/week. Her treatment for multiple sclerosis is not known to be associated with bone metabolism and thus cannot be held responsible for playing a role in the pathogenesis of her stress fractures. Bazelier et al. found that multiple sclerosis itself is associated with an increased risk of osteoporotic fractures—especially hip fractures—mainly resulting from an increased risk of falls, particularly when taking glucocorticoids or antidepressants [33]. However, in our case, the patient sustained no fall, had a normal osteoporotic workup, and took no glucocorticoid or antidepressant medication.

## 5. Conclusion

Although internal fixation remains the gold standard in nondisplaced femoral neck stress fractures, femoral osteotomy and THA must be part of the treatment options. Total hip arthroplasty, especially with the advances in material technology, tribology, and surgical technique, has excellent clinical outcomes and high patient satisfaction rates, making it now a more interesting avenue for young and active patients. Thus, when it comes to femoral neck stress fractures, it should be considered in cases of failed internal fixation or in those cases not responding to conservative treatment.

## Figures and Tables

**Table 1:** Case series of bilateral femoral neck fractures with their treatment, failure rates, and follow-up duration.

Author	Tensile/compressive	Treatment	Failure/treatment	Follow-up	Patient age/sex
Naik et al.					
Case 1 Case 2 Case 3 Case 4	L: n.a.; R: 1 L: 1; R: 3 L: 3; R: 1 L: 2; R: 3	L: OS screw; R: OT L: OS screw; R: OT L: OT; R: OT L: OT; R: OT	L: HA No No No	28 m 12 m 6 m 7 y	38 M 38 F 48 F 40 F
Selek et al.					
Case 1 Case 2 Case 3	L: n.a.; R: n.a. L: n.a.; R: n.a. L: n.a.; R: n.a.	L: OS screws; R: OS screws L: OS screws; R: OS screws L: OS screws; R: OS screws	No No No	4 y 28 m 2 y	30 F 35 F 30 F
Moo et al.					
Case 1 Case 2 Case 3	L: 2; R: 1 L: 1; R: 3 L: n.a.; R: 2	L: OS screws; R: cons L: cons; R: OS screws L: OS screws; R: OS screws	No No No	28 m 4 m 24 m	19 M 18 M 20 M
Cakmak et al.	L: 2; R: 3	L: OS screws; R: HA	No	18 m	82 F
Nagao et al.	L: 2; R: 3	L: OS screws; R: cons	L: HA; R: HA	24 m	36 M
Hernigou et al.	L: 1; R: 1	L: OS DHS; R: OS DHS	No	2 w	38 F
Vaishya et al.	L: 1; R: 1	L: OS screws; R: OS screws	No data	No data	50 M
Santoso et al.	L: 1; R: 1	L: cons; R: cons	No	5 m	37 M
Oliveira et al.	L: 1; R: 1	L: OS; R: OS	No data	No data	43 M
Sariyilmaz et al.	L: 1; R: 1	L: VOT (blpl); R: VOT (blpl)	No	2 y	26 F
Baki et al.	L: 1; R: 1	L: OS screws; R: OS screws	No	6 m	22 F
Nemoto et al.	L: 1; R: 1	L: OS screws; R: OS screws	No	2 y	24 M
Webber et al.	L: 1; R: 1	L: OS screws; R: OS screws	No	No data	23 M
Wright et al.	L: 3; R: 3	L: OS screws; R: OS screws	No access to full article	No access	24 M
Bouchoucha et al.	L: 3; R: 2	L: VOT (blpl) R: OS (DHS)	No data	No data	15 F
Naranje et al.	L: 3; R: 2	L: OS screws; R: OS screws	No	12 m	34 M
Carpintero et al.	L: 2; R: 3	L: OS gamma; R: OS gamma	R: AVN L: AVN/THA	No data	32 M
Khadabadi et al.	L: n.a.; R: n.a.	L: OS DHS; R: OS DHS	No	12 m	25 M
Romero et al.	L: 2; R: 1	L: OS screws; R: OS screws	No	6 m	19 M
Chameseddine et al.	L: 3; R: 3	L: HA; R: HA	No	6 m	71 F
Pankaj et al.	L: 2; R: 3	R: HA; R: OS screws	No	±5 y	58 M
Haddad et al.	L: 3; R: n.a.	L: OS screws; R: OS screws	L: delayed union/conservative	4 m	56 F
Mariani et al.	L: 3; R: 3	L: THA; R: THA	No	24 m	24 M
Eberle et al.	L: 2; R: 1	L: cervical nail (“three-flanged nail”); R: cons	No	40 m	54 F
Zuckerman et al.	L: 1; R: 1	L: OS DHS; R: OS DHS	No	24 m	46 F
Voss et al.	L: 1; R: 1	L: cons; R: OS screws	R/L: nonunion	24 m	30 F
Bailie et al.	L: 1; R: 1	L: cons; R: cons	No	2m	15 M
Annan et al.	L: n.a.; R: n.a.	L: DHS + VOTR: DHS + VOT	No	6 m	29 F
Ichikawa et al.	L: 2; R: 2	L: OS screws; R: OS screws			61 F
Chouhan et al.	L: 1; R: 1	L: cons; R: cons	No	6 m	32 M
Hootkani et al.	L: 3; R: 3	L: HA; R: HA	No	24 m	28 M
Scheerlinck et al.	L: 1; R: 1	L: cons; R: cons	L: displaced fracture after accidental fall/OS by 2 screws	14 w	8 F
Kharazzi et al.	n.a.	L: OS pins; R: OS pins	No	15 y	22 M
Rengman	n.a.	L: cons; R: cons	No	No data	21 M
Vento et al.	n.a.	L: OS pins; R: OS pins & DHS	No data	No data	53 M
Miller	L: 1; R: 1	L: cons; R: cons	No	8 m	36 F
Slipman et al.	L: 1; R: 1	L: cons; R: cons	No data	No data	36 F
Kalaci et al.	L: 1; R: 1	L: OS screws; R: OS screws	No	6 w	18 F
Gurdezi et al.	L: 1; R: 1	L: cons; R: cons	No	12 m	61 F
*N* total	Type 1: 41 (56%) Type 2: 13 (18%) Type 3: 19 (26%) Total known: 73 Total unknown: 21	% of prosthesis Cons: 21 (23%) OS: 52 (56%) OT: 11 (12%) Arthroplasty (6HA, 2THA): 8 (9%) Total known: 92 Total unknown: 2	% of failure 2 screws: HA 1 screw: delayed union 1 screw: nonunion 1 cons: nonunion 1 cons: HA 1 cons: OS 1 nail: AVN 1 nail: THA	Mean: 23, 4 m	Mean: 35 y Mean M: 30 y Mean F: 40 y

L: left hip; R: right hip; OS: osteosynthesis; HA: hemiarthroplasty; OT: osteotomy; cons: conservative treatment; DHS: dynamic hip screw; VOT: valgus osteotomy; blpl: blade plate; THA: total hip arthroplasty; NS: no score; AVN: avascular necrosis; Type 1: compression fracture; type 2: tension fracture; type 3: displaced fracture; n.a.: not available.
